# Overload of anxiety on postural control impairments in chronic stroke survivors: The role of external focus and cognitive task on the automaticity of postural control

**DOI:** 10.1371/journal.pone.0252131

**Published:** 2021-07-22

**Authors:** Zahra Ghorbanpour, Ghorban Taghizadeh, Seyed Ali Hosseini, Ebrahim Pishyareh, Farhad Tabatabai Ghomsheh, Enayatollah Bakhshi, Hajar Mehdizadeh

**Affiliations:** 1 Department of Occupational Therapy, School of Rehabilitation Sciences, University of Social ‎Welfare and Rehabilitation Sciences‏ (USW‏R), Tehran, Iran; 2 Department of Occupational Therapy, Rehabilitation Research Center, School of Rehabilitation Sciences, Iran University of Medical Sciences, Tehran, Iran; 3 Pediatric Neurorehabilitation Research Center, University of Social ‎Welfare and Rehabilitation Sciences‏ ‎‎(USW‏R), Tehran,Iran; 4 Department of Biostatistics and Epidemiology, University of Social ‎Welfare and Rehabilitation Sciences‏ ‎‎(USW‏R), Tehran,Iran; 5 Department of Neurosciences, School of Advanced Technologies in Medicine, Tehran University of Medical Sciences (TUMS), Tehran, Iran; Texas State University, UNITED STATES

## Abstract

**Background:**

Despite the high prevalence of anxiety among chronic stroke survivors and evidence of its negative effects on postural control in healthy subjects, it is unclear whether anxiety also affects postural control in these patients. Recent evidence of improved postural control of healthy subjects by distracting the attention using an external focus (EF) or cognitive task, raises the question of whether similar benefits would be observed in stroke survivors. Thus, the current study aimed to investigate the effects of anxiety and distracting the attention on postural control of chronic stroke survivors in terms of both postural sway measures and neuromuscular regulation.

**Methods:**

Postural sway measures and ankle muscle activity of chronic stroke survivors with the high and low level of anxiety (HA-stroke (n = 17), and LA-stroke (n = 17), respectively) and age-, sex-, height-, and weight-matched healthy subjects (n = 17) were assessed while standing on rigid and foam surfaces under following conditions: baseline, internal focus (IF), EF, simple and hard cognitive tasks (SC and HC, respectively).

**Results:**

Stroke survivors, particularly HA-stroke participants, showed greater postural sway measures (i.e. postural instability) and enhanced co-contraction of ankle muscles (i.e. stiffening of the neuromuscular system) compared with healthy subjects. As opposed to baseline and IF conditions, postural instability and neuromuscular stiffening significantly reduced in EF condition and decreased more in cognitive task conditions, particularly HC condition.

**Conclusions:**

The results suggest that anxiety enhances stroke-induced postural instability promoting improper neuromuscular control of posture with stiffening strategy, which can be alleviated by EF and cognitive tasks.

## Introduction

Postural control of standing is critical for doing functional activities, which underlie many activities of daily living [1]. Stroke survivors experience different sensory and motor impairments (e.g. decreased proprioception, over-reliance on visual inputs, muscle activation deficits, etc.), leading to impairments of postural control [2]. As a consequence of impaired postural control, about 40–70% of stroke survivors experience a fall each year, leading to fear of falling [3], which hampers activities of daily living and social participation and decreases the quality of life [3, 4]. Postural control requires a precisely coordinated activation of the neuromuscular system, which depends on the integration of visual, somatosensory, and vestibular information [5]. Although postural control is highly automatic and is efficiently done without conscious control in most situations [6], dual-task studies have indicated that it requires attentional resources that enhances with aging [7], impairments of the central nervous system such as stroke, and the task difficulty [8]. To compensate for the decreased capabilities of sensory and motor processing to operate in an efficient automatic manner, stroke survivors use excessive attentional resources for controlling posture (i.e. conscious postural control), leading to decreased automaticity of postural control and increased postural instability [8, 9].

In addition to impaired postural control, one of the most common critical complications of the stroke in both acute and chronic phases is anxiety with a prevalence of 18–25% [10] and debilitating consequences such as decreased quality of life, daily functioning, and social interactions [11]. The negative effects of anxiety on postural control have been reported previously in healthy subjects and neurological disorders such as Parkinson’s disease [5, 12–15] and explained by the neural connections between areas of the brain responsible for emotional control and areas controlling posture and balance [16]. Further, anxiety may affect the processing of visual information [12] and the interactions between the visual, vestibular, and somatosensory inputs as key elements of the postural control system [5]. Moreover, as stated by “attentional control theory”, a high level of anxiety leads to an attentional bias to task-unrelated/threat-related stimuli [17, 18], which in turn results in decreased attentional resources allocated to the task of hand and impairments in performance [17]. To compensate for this attentional bias, individuals with anxiety may use an alternative processing strategy (i.e. directing attention to movement control or internal focus (IF)) [17, 19, 20], which may help alleviate anxiety. However, based on the constrained action hypothesis, paying attention to highly automatic movement processes may impair task performance [21] that has been reported for postural control in various conditions [22]. Thus, anxiety may exacerbate decreased automaticity of postural control, which is found in stroke survivors. However, despite the high prevalence of anxiety among stroke survivors and recent evidence regarding the negative effects of anxiety on postural control, the question remains whether anxiety can affect postural control in stroke survivors.

Recent studies indicated that withdrawing attention from postural control (e.g. by focusing the attention on an external cue (i.e. external focus (EF)) or performing a concurrent cognitive task while standing) results in improved postural control/stability in both healthy young and older adults as evidenced by decreased postural sway measures [23–27]. Based on the constrained action hypothesis, it has been suggested that withdrawing attention away from postural control using EF and cognitive task enables more efficient postural control due to the unconstrained function of automatic processes, leading to improved postural stability [23, 26, 27]. Another possible mechanism for improving postural control in such conditions is the use of stiffening strategy (e.g. enhanced muscle activity at the ankle joint) to maximize the stability and reduce the allocation of attentional resources to postural control [28, 29]. Conversely, focusing the attention inwardly on motor control (i.e. IF), as occurred in neurological disorders such as stroke and individuals with a high level of anxiety, impede automatic processes by promoting a conscious mode of motor control [22, 30]. Previous studies have reported contradictory findings regarding the effects of cognitive task on postural control of stroke survivors. While some studies reported reduced postural stability of stroke survivors [31, 32], others reported inconsistent evidence of postural instability [33] or improved postural stability [9, 34] by performing a concurrent cognitive task. Conflicting results have also been found regarding the effects of EF on motor control in stroke survivors, with some studies reporting improvement [35–37] and other deterioration [38] of motor control during different tasks such as gait [37], body weight shifting [36], reach and grasp [35] and stepping [38]. However, to the best of our knowledge, no studies so far have investigated the effects of EF on standing postural control in chronic stroke survivors. Furthermore, the mechanism of the effects of anxiety, as well as EF and cognitive task (i.e. promoting stiffening strategy vs. automaticity) on postural control of chronic stroke survivors, has not yet been investigated from neuromuscular control perspectives (e.g. using electromyography (EMG)). Identifying the mechanism that underlies the effects of anxiety, EF and cognitive task on postural control in chronic stroke survivors may open the way for developing effective therapeutic interventions aimed at improving postural control and decreasing risk of falls in these patients.

Thus, the purpose of the current study was twofold: first, to determine the effects of anxiety on postural control of chronic stroke survivors while standing on stable and unstable surfaces in terms of both postural sway measures and the neuromuscular regulation of balance (i.e. EMG of ankle muscles) and second, to compare the effects of withdrawing attention by EF and cognitive task on postural control among chronic stroke survivors with low and high levels of anxiety and age-, sex-, height-, and weight-matched healthy subjects.

## Materials and methods

### Participants

Thirty-four chronic stroke survivors (17 stroke patients with a high level of anxiety (HA-stroke) and 17 stroke survivors with a low level of anxiety (LA-stroke)), as well as 17 sex-, age-, and height-, and weight-matched healthy control participated in this study. The main inclusion criteria for the stroke survivors included the first-ever stroke in the middle cerebral artery that neuro-radiologically confirmed with the onset of > 6 months; ability to stand alone and walk without any assistance for a distance of at least 10 meters; absence of unilateral neglect (i.e. Star Cancelation Test score ≥ 44) [39] and ability to complete the most difficult experimental condition (i.e. quiet standing on foam surface for 70 s). Healthy control subjects were included if they didn’t have anxiety (i.e. score equal or less than 7 on the anxiety subscale of Hospital Anxiety and Depression Scale (HADS-A)) [40]. Exclusion criteria for either group were as follows: neurologic disorders (except stroke for stroke groups) or musculoskeletal disorders such as low back pain, flat foot, a recent history of lower limb fracture, recent surgical operations in the spine or lower extremity; cognitive problems (i.e. score <23 on the Mini-Mental Status Examination) [41]; depression (i.e. score > 7 on the of HADS-Depression subscale (HADS-D)) [42], diabetes, vestibular disorders, vertigo, pain, and visual problems that not corrected by glasses. None of the participants used any medications that could affect postural control. All participants completed the Geriatric Anxiety Inventory (GAI) [43], HADS-A and Beck Anxiety Inventory for evaluating anxiety, HADS-D, and Beck Depression Inventory [42] for measuring depression, Montreal Cognitive Assessment [41] and Mini-Mental Status Examination for assessing cognitive function, Fatigue Severity Scale [44, 45] for assessing fatigue severity; and Visual Analogue Scale for scoring pain. The number of falls during the past year was also recorded. Based on the scores on the GAI and HADS-A, stroke survivors were divided into LA-stroke (n = 17, who obtained scores < 9 on the GAI and < 11 on the HADS-A) and HA-stroke (n = 17, who obtained scores ≥ 9 on the GAI and ≥ 11 on the HADS-A). The participants in the LA- and HA-stroke groups were matched based on age, sex, height, weight, location of stroke lesion, and paretic side. The HA-stroke group did not report consuming different medications (to manage anxiety) compared with the LA-stroke group. The ethics committee of the University of Social Welfare and Rehabilitation Sciences approved the study (IR.uswr.Rec.1397.010) and all participants provided written informed consent before the study.

### Experimental procedure

#### Postural performance

The postural performance was evaluated by measuring the center of pressure (COP) sway using a Kistler force plate (Kistler, Winterthur, Switzerland) at a sampling frequency of 100 Hz. The participants were asked to quietly stand barefoot in a bipedal straight position, arms alongside the trunk and their feet close together, on the rigid surface of the force plate or 10.5 cm thick foam placed on the force plate under five experimental conditions: baseline, EF, IF, simple cognitive task (SC), and hard cognitive task (HC). The conditions were performed in a randomized order. Participants were asked to look straight ahead during all conditions. Each condition was performed twice (i.e. two trials), and each trial lasted 70 seconds. To prevent fatigue, one and five minutes of rest interval was considered between trials and experimental conditions, respectively. In the baseline condition, participants were required to quietly stand as described above. In the EF condition, two rectangular paper (30.5×17cm) were put on the force plate or foam (one under each foot) and the participants were asked to mentally focus on these papers without looking at them [46]. In the IF condition, the participants were instructed to mentally concentrate on their feet without looking at them. To confirm that attention was properly allocated during both the EF and IF conditions, the subjects were requested to grade the percentage of attention they had allocated to the task. If the reported grade was ≤ 50%, the trial was repeated.

The cognitive task was a backward digit span with two levels of difficulty (simple and hard), as described previously, which was determined based on the maximum backward digit span of each subject [47]. In brief, the participants were asked to listen carefully to a series of random digits before starting the COP recording, mentally repeat the digits in reverse order while the COP data were recorded, and verbally report the digits after the accomplishment of COP recording. The number of digits presented in the HC condition was equal to the maximum backward digit span plus one while half of the digits of the HC condition were presented in the SC condition. There were three types of error (omission, wrong number, and order error) in cognitive tasks. If the error was greater than 1 in the SC condition and greater than 2 in the HC condition, the trial was repeated.

#### Electromyography activity

During the above-mentioned experimental conditions, muscle activity of the ankle muscles (i.e. tibialis anterior (TA) and medial gastrocnemius (MGA)) was recorded using surface EMG (Myon EMG, Switzerland) at a sampling frequency of 1000 Hz. EMG electrodes were put on the skin surface over the TA and MGA muscles of both sides. The maximal voluntary isometric contraction (MVC) of the TA [48] and MGA [49] was obtained to normalize the EMG activity (%MVC) during the experimental conditions.

### Data analysis

To assess postural control in different experimental conditions, COP sway data was used to calculate the path length, mean velocity, and SD of velocity along the anterior-posterior (AP) and medial-lateral (ML) directions. These COP measures were selected because of their well-confirmed reliability for assessing postural performance in subjects with pathologies and healthy subjects and their higher values indicate postural instability [50]. The original raw EMG data obtained from ankle muscles were band-pass filtered at 20–450 Hz. The root-mean-square (RMS) of the filtered EMG data was calculated as a percentage of the EMG value during the MVC. The normalized RMS of the TA and MGA muscles were then used for calculating the averaged co-contraction level of these muscles based on the following formula, which was described previously [51, 52]: Co-contraction index (CCI) = $\frac{{2I}_{antagonist}}{I_{total}}$ × 100.

### Statistical analysis

A prior power analysis (type I error probability = 0.05, type II error probability (statistical power) = 0.20, dropout rate = 10%), which was done for the SD of velocity along ML direction obtained in a pilot study showed that 17 subjects were necessary for each group. The Shapiro-Wilk test confirmed the normal distribution of both postural sway measures and CCI data. The mean value of two trials of the same condition was evaluated for each postural sway measure and CCI. The main and interaction effects of standing surfaces and conditions on different postural sway measures and CCI in the LA-stroke, HA-stroke, and healthy control groups were analyzed using a 3 × 2 × 5 (group × standing surface × condition) three-way repeated measure analysis of variance (ANOVA) with a critical α level of 0.05. The effect size of both main effects and interaction effects was determined by calculation of $\eta_{p}^{2}$. Significant three-way interactions were followed up with simple effects testing. Then, multiple comparisons were done using the Bonferroni adjustment method if significant main effects were found. The Bonferroni-adjusted P value for multiple comparisons was considered as P<0.0005.

## Results

### Participants

Seventeen HA-stroke survivors (8 female, 9 male) by mean ± SD age of 49.7 ± 8.73 years, 17 LA-stroke survivors (8 female, 9 male) by mean ± SD age of 52.82 ± 13.61 years and 17 age-, sex-, height-, and weight-matched healthy control subjects (8 female, 9 male) by mean ± SD age of 51.64 ± 10.15 years participated in the study. There was no difference among the three groups, except with their scores on the HADS-A, Beck Anxiety Inventory, and GAI (Table 1). The results found a significantly higher level of anxiety based on HADS-A, Beck Anxiety Inventory, and GAI scores in the HA-stroke group compared with the LA-stroke and healthy control groups.

**Table 1:** Demographic characteristics of the participants in each group.

Variable	Healthy Control Group (n = 17)	LA-stroke Group (n = 17)	HA-stroke Group (n = 17)	P
Sex (female/male)	8/9	8/9	8/9	-
Etiology (ischemia/hemorrhage)	-	13/4	12/5	0.7
Affected side (right/left)	-	7/10	7/10	-
Age (years)	51.64 ± 10.15	52.82 ± 13.61	49.7 ± 8.73	0.7
Body mass index (Kg/m^2^)	25.35 ± 3.8	25.72 ± 3.9	27.71 ± 6.4	0.32
Mini Mental Status Examination	28.29 ± 1.96	27.11 ± 2.31	26.70 ± 2.23	0.10
Montreal Cognitive Assessment	26.29 ± 1.53	25.94 ± 2.24	25.17 ± 1.74	0.21
HADS^†^-Depression subscale	3.47 ± 1.90	3.23 ± 2.30	4.05 ± 1.43	0.44
HADS-Anxiety subscale	2.88 ± 2.66	3.17 ± 2.81	12.17 ± 2.15‡§	***<0*.*001***
Beck Depression Inventory	3.64 ± 2.69	3.40 ± 2.03	4.00 ± 1.87	0.78
Beck Anxiety Inventory	3.94 ± 3.49	6.35 ± 5.08	29.17 ± 8.23‡§	***<0*.*001***
Geriatric Anxiety Inventory	3.11 ± 2.14	2.35 ± 2.47	11.76 ± 2.35‡§	***<0*.*001***
Fatigue Severity Scale	22.17 ± 9.74	25.29 ± 10.12	28.29 ± 7.83	0.16

^†^ Hospital Anxiety and Depression Scale

‡ indicates a significant difference compared with the healthy control group

§ indicates a significant difference compared with the LA-stroke group

### Postural performance

**Fig 1:**
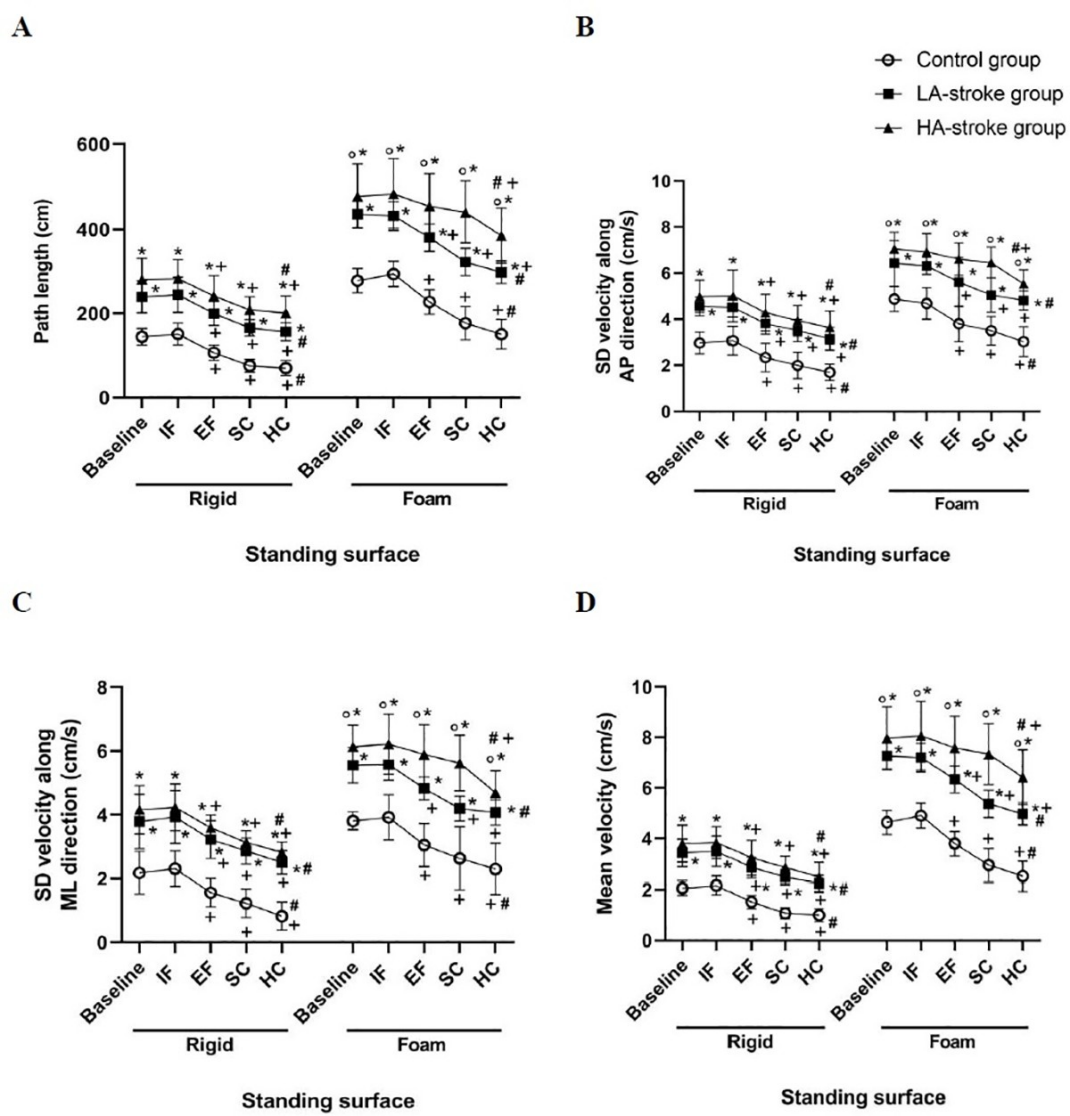
The interaction effect of the group, standing surface, and condition on different postural sway measures: Path length (A), SD of velocity along anterior-posterior (AP) direction (B), SD of velocity along medial-lateral (ML) direction (C), and mean velocity (D). *P<0.0005 compared with the control group in the same condition, °P<0.0005 compared with the LA-stroke group in the same condition, +P<0.0005 compared with the baseline condition in the same group, and #P<0.0005 in comparison of hard cognitive task (HC) condition with other conditions in the same group. *It should be noted that*, *in the three groups*, *all postural sway measures were significantly greater while standing on a foam surface compared with standing on a rigid surface* (IF: Internal focus; EF: External focus; SC: Simple cognitive task).

The descriptive data of postural sway measures are presented in Table 2. The results showed a significant main effect of group, standing surface, and condition on all postural sway measures. All two-way interaction effects were also significant for different postural sway measures, except group × condition interaction for SD of velocity along AP direction (F = 1.84, P = 0.07, $\eta_{p}^{2}$ = 0.07), as well as group × standing surface and condition × standing surface interactions (F = 2.71, P = 0.07, $\eta_{p}^{2}$ = 0.10 and F = 0.91, P = 0.46, $\eta_{p}^{2}$ = 0.02, respectively) for SD of velocity along ML direction (Table 3). The three-way interaction of group × condition× standing surface was statistically significant for all postural sway measures (Table 2). The analyses of the simple main effects following significant three-way interaction for each postural sway measure indicated that the inter-groups differences were significant in all combinations of standing surfaces (i.e. rigid and foam) and conditions (i.e. baseline, IF, EF, SC, and HC). Moreover, the results of simple main effects analysis indicated that inter-conditions differences regarding different postural sway measures were significant in all combinations of groups (i.e. control, LA-stroke, and HA-stroke) and standing surfaces (i.e. rigid and foam) (Table 4). The results of multiple comparisons showed that postural sway measures in the three groups (i.e. control, LA-stroke, and HA-stroke groups) were significantly higher while standing on a foam surface than standing on a rigid surface in different conditions including baseline, IF, EF, SC, and HC. Further, during standing on both rigid and foam surfaces in different conditions (baseline, IF, EF, SC, and HC), the postural sway measures were significantly greater in both HA-stroke and LA-stroke groups as opposed to the healthy group. However, only during standing on a foam surface, a significant difference was found between the LA-stroke and HA-stroke groups. During standing on the rigid surface, EF and both SC and HC resulted in a significant decrease of different postural sway measures in the three groups. During standing on the foam surface, EF and both SC and HC resulted in a significant decrease of different postural sway measures in the control and LA-stroke group. However, in the HA-stroke group, a significant decrease of postural sway measures was only observed in the HC condition during standing on the foam surface. The greatest decrease of the postural sway measures was found in the HC condition while standing on both rigid and foam surfaces (Fig 1A–1D).

**Table 2:** Descriptive data (mean ± SD) for postural sway measures and co-contraction index (CCI) of the tibialis anterior and medial gastrocnemius muscles in different conditions.

Condition	Baseline			Internal focus			External focus			Simple cognitive task			Hard cognitive task		
Group	Control	LA-Stroke	HA-stroke	Control	LA-Stroke	HA-stroke	Control	LA-Stroke	HA-stroke	Control	LA-Stroke	HA-stroke	Control	LA-Stroke	HA-stroke
**Standing on rigid surface**															
**Path length (cm)**	144.56 ± 20.05	239.91 ± 38.21	280.67 ± 51.07	151.20 ± 25.99	244.70 ± 42.27	283.38 ± 44.47	106.61 ± 18.05	200.06 ± 28.58	241.59 ± 48.54	75.68 ± 15.30	165.81 ± 19.68	208.77 ± 31.31	70.39 ± 17.99	156.52 ± 21.59	201.45 ± 41.22
**SD of Velocity (A.P) (cm/s)**	2.97 ± 0.47	4.56 ± 0.41	4.99 ± 0.71	3.07 ± 0.62	4.51 ± 0.42	5.01 ± 0.75	2.34 ± 0.62	3.91 ± 0.46	4.29 ± 0.79	1.99 ± 0.58	3.71 ± 0.46	4.03 ± 0.67	1.49 ± 0.36	3.25 ± 0.50	3.65 ± 0.72
**SD of Velocity (M.L) (cm/s)**	2.18 ± 0.68	3.89 ± 0.85	4.36 ± 0.76	2.32 ± 0.56	3.93 ± 0.83	4.44 ± 0.73	1.56 ± 0.45	3.23 ± 0.58	3.79 ± 0.40	1.22 ± 0.45	2.67 ± 0.41	3.34 ± 0.36	0.83 ± 0.44	2.52 ± 0.37	3.03 ± 0.33
**Mean velocity (cm/s)**	2.07 ± 0.29	3.43 ± 0.55	4.01 ± 0.73	2.16 ± 0.37	3.50 ± 0.60	4.05 ± 0.64	1.52 ± 0.26	2.86 ± 0.41	3.45 ± 0.69	1.08 ± 0.22	2.37 ± 0.28	2.98 ± 0.45	1.01 ± 0.26	2.24 ± 0.31	2.88 ± 0.59
**CCI of paretic limb (%)**	19.56 ± 4.57	48.79 ± 7.06	50.09 ± 5.38	21.55 ± 5.08	49.55 ± 7.51	51.81 ± 5.60	19.69 ± 4.24	42.05 ± 6.35	44.59 ± 4.93	17.81 ± 4.00	37.36 ± 5.82	41.59 ± 5.34	17.07 ± 3.26	32.68 ± 5.48	35.03 ± 5.18
**CCI of non-paretic (%)**	20.38 ± 3.99	53.66 ± 6.43	57.78 ± 7.04	21.51 ± 3.97	54.06 ± 6.75	58.22 ± 7.07	18.99 ± 3.71	47.64 ± 6.46	51.69 ± 6.38	17.89 ± 3.66	45.06 ± 6.75	48.13 ± 6.24	16.38 ± 3.23	41.56 ± 6.71	44.72 ± 6.26
**Standing on foam surface**															
**Path length (cm)**	278.23 ± 29.21	436.37 ± 31.56	478.19 ± 75.26	294.24 ± 30.15	432.31 ± 34.54	484.04 ± 81.96	227.61 ± 29.16	380.69 ± 32.42	455.07 ± 75.92	177.05 ± 39.89	322.54 ± 32.76	440.59 ± 72.70	150.83 ± 35.58	298.07 ± 26.64	385.47 ± 65.73
**SD of Velocity (A.P) (cm/s)**	4.58 ± 0.63	6.43 ± 1.00	6.65 ± 0.72	4.69 ± 0.69	6.32 ± 0.37	6.73 ± 0.80	3.80 ± 0.78	5.62 ± 1.07	6.33 ± 0.70	3.50 ± 0.61	5.06 ± 0.74	6.13 ± 0.68	3.03 ± 0.65	4.82 ± 0.42	5.34 ± 0.62
**SD of Velocity (M.L) (cm/s)**	3.81 ± 0.28	5.56 ± 0.56	6.14 ± 0.68	3.92 ± 0.70	5.58 ± 0.50	6.22 ± 0.95	3.06 ± 0.68	4.83 ± 0.37	5.90 ± 0.94	2.63 ± 1.00	4.20 ± 0.38	5.63 ± 0.88	2.30 ± 0.81	4.08 ± 0.39	4.70 ± 0.69
**Mean velocity (cm/s)**	4.64 ± 0.49	7.27 ± 0.53	7.97 ± 1.25	4.90 ± 0.50	7.21 ± 0.58	8.07 ± 1.37	3.79 ± 0.49	6.34 ± 0.54	7.58 ± 1.27	2.95 ± 0.66	5.38 ± 0.55	7.34 ± 1.21	2.51 ± 0.59	4.97 ± 0.44	6.42 ± 1.10
**CCI of paretic limb (%)**	39.32 ± 4.37	75.59 ± 8.83	82.11 ± 7.00	40.43 ± 4.45	76.04 ± 8.98	81.64 ± 7.62	32.36 ± 3.31	64.04 ± 8.69	74.58 ± 6.94	27.94 ± 3.16	61.57 ± 8.45	71.12 ± 6.88	25.17 ± 2.72	52.23 ± 7.68	66.41 ± 6.41
**CCI of non-paretic (%)**	40.55 ± 3.75	80.79 ± 6.14	86.70 ± 8.11	39.88 ± 3.49	79.41 ± 6.34	87.13 ± 9.07	33.21 ± 3.71	70.95 ± 6.04	80.15 ± 7.60	28.77 ± 3.54	65.32 ± 5.94	76.06 ± 6.82	24.51 ± 3.40	60.35 ± 5.84	68.35 ± 6.16

**Table 3:** Summary of analysis of variance of different postural sway measures: F ratios, P values, and effect sizes by variable.

	Path length (cm)			SD of velocity (A.P) (cm/s)			SD of velocity (M.L) (cm/s)			Mean velocity (cm/s)		
	F	P	$\eta_{p}^{2}$	F	P	$\eta_{p}^{2}$	F	P	$\eta_{p}^{2}$	F	P	$\eta_{p}^{2}$
**Main effect**												
Group	156.43	***<0*.*001***	0.99	90.40	***<0*.*001***	0.79	173.09	***<0*.*001***	0.88	156.43	***<0*.*001***	0.99
Condition	424.75	***<0*.*001***	0.90	261.02	***<0*.*001***	0.84	344.53	***<0*.*001***	0.88	424.75	***<0*.*001***	0.90
Standing surface	538.63	***<0*.*001***	0.92	531.30	***<0*.*001***	0.92	225.13	***<0*.*001***	0.82	538.63	***<0*.*001***	0.92
**Interaction effect**												
Group × Condition	5.49	***<0*.*001***	0.19	1.84	0.07	0.07	2.86	***0*.*005***	0.11	5.49	***<0*.*001***	0.19
Group × Standing surface	13.32	***<0*.*001***	0.36	4.67	***<0*.*001***	0.16	2.71	0.07	0.10	13.32	***<0*.*001***	0.36
Condition × Standing surface	16.55	***<0*.*001***	0.26	2.59	***0*.*04***	0.05	0.91	0.46	0.02	16.55	***<0*.*001***	0.26
Group × Condition × Standing surface	5.31	***<0*.*001***	0.18	2.82	***0*.*006***	0.11	3.20	***0*.*002***	0.12	5.31	***<0*.*001***	0.18

**Table 4:** Simple main effects for analyzing inter-groups and inter-conditions differences of postural sway measures for all combinations.

			Path length		SD of velocity (A.P) (cm/s)		SD of velocity (M.L) (cm/s)		Mean velocity	
			(cm)						(cm/s)	
			F	P	F	P	F	P	F	P
**Simple effect for analyzing inter-groups differences**										
**Standing surface**	**Rigid**	Baseline	45.46	***<0*.*001***	44.60	***<0*.*001***	53.73	***<0*.*001***	45.46	***<0*.*001***
		IF	43.03	***<0*.*001***	39.99	***<0*.*001***	51.60	***<0*.*001***	43.03	***<0*.*001***
		EF	44.52	***<0*.*001***	42.26	***<0*.*001***	56.80	***<0*.*001***	44.52	***<0*.*001***
		SC	42.98	***<0*.*001***	47.50	***<0*.*001***	48.97	***<0*.*001***	42.98	***<0*.*001***
		HC	41.32	***<0*.*001***	51.86	***<0*.*001***	56.00	***<0*.*001***	41.32	***<0*.*001***
	**Foam**	Baseline	103.63	***<0*.*001***	50.98	***<0*.*001***	61.72	***<0*.*001***	103.63	***<0*.*001***
		IF	89.70	***<0*.*001***	46.13	***<0*.*001***	59.05	***<0*.*001***	89.70	***<0*.*001***
		EF	125.30	***<0*.*001***	66.95	***<0*.*001***	86.48	***<0*.*001***	125.30	***<0*.*001***
		SC	162.34	***<0*.*001***	68.84	***<0*.*001***	94.40	***<0*.*001***	162.34	***<0*.*001***
		HC	130.92	***<0*.*001***	57.76	***<0*.*001***	64.85	***<0*.*001***	130.92	***<0*.*001***
**Simple effect for analyzing inter-conditions differences**										
**Group**	**Control**	Rigid	13.16	***<0*.*001***	17.41	***<0*.*001***	16.71	***<0*.*001***	13.16	***<0*.*001***
		Foam	35.94	***<0*.*001***	19.79	***<0*.*001***	21.27	***<0*.*001***	35.94	***<0*.*001***
	**LA-stroke**	Rigid	15.47	***<0*.*001***	12.16	***<0*.*001***	17.16	***<0*.*001***	15.47	***<0*.*001***
		Foam	36.68	***<0*.*001***	20.71	***<0*.*001***	21.64	***<0*.*001***	36.68	***<0*.*001***
	**HA-stroke**	Rigid	13.86	***<0*.*001***	14.09	***<0*.*001***	16.03	***<0*.*001***	13.86	***<0*.*001***
		Foam	14.49	***<0*.*001***	12.32	***<0*.*001***	15.88	***<0*.*001***	14.49	***<0*.*001***

IF, Internal focus; EF, External focus; SC, Simple cognitive task; HC, Hard cognitive task

### EMG activity

**Fig 2:**
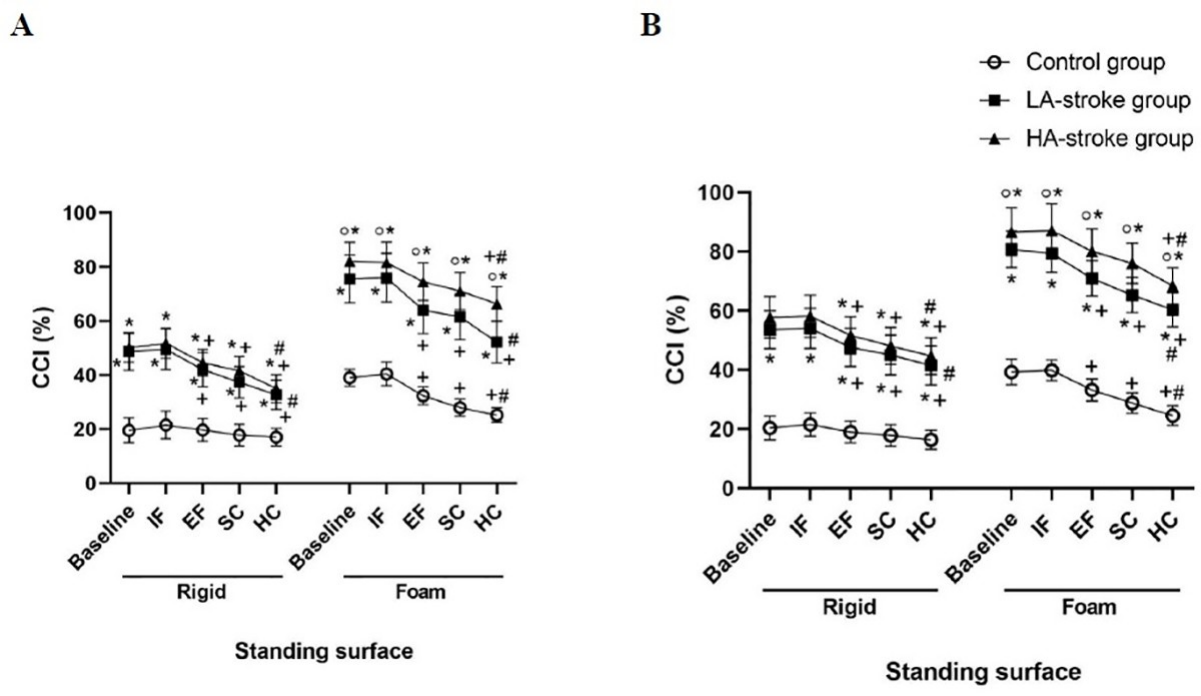
The interaction effect of the group, standing surface, and condition on the co-contraction index (CCI) of the tibialis anterior and medial gastrocnemius muscles: (A) paretic limb and (B) non-paretic limb of LA- and HA-stroke groups. *P<0.0005 compared with the control group in the same condition, °P<0.0005 compared with the LA-stroke group in the same condition, +P<0.0005 compared with the baseline condition in the same group, and #P<0.0005 in comparison of hard cognitive task (HC) condition with other conditions in the same group. *It should be noted that*, *in the three groups*, *the CCI was significantly greater while standing on a foam surface compared with standing on a rigid surface* (IF: Internal focus; EF: External focus; SC: Simple cognitive task).

The descriptive data of CCI are presented in Table 2. The results revealed a significant main effect of group, standing surface, and condition as well as their significant interaction effects on the CCI (Table 5). The analyses of the simple main effects following significant three-way interaction of group × standing surface × condition for CCI indicated that the inter-groups differences were significant in all combinations of standing surfaces (i.e. rigid and foam) and conditions (i.e. baseline, IF, EF, SC, and HC). Moreover, the results of simple main effects analysis indicated that inter-conditions differences regarding CCI were significant in all combinations of groups (i.e. control, LA-stroke, and HA-stroke) and standing surfaces (i.e. rigid and foam), with the exception of combination of control group and rigid surface (Table 6). The results of multiple comparisons indicated that the CCI of both paretic and non-paretic limbs was significantly higher in both the LA-stroke and HA-stroke groups compared to the control group during standing on both rigid and foam surfaces in different conditions (i.e. baseline, IF, EF, SC, and HC). Besides, only during standing on a foam surface, the HA-stroke group showed higher CCI of both paretic and non-paretic limbs than did the LA-stroke group. Standing on a foam surface resulted in a significant increase of the CCI in the three groups. During standing on foam and rigid surfaces, EF and both SC and HC resulted in a significant reduction of the CCI of both paretic and non-paretic limbs as opposed to the baseline and IF conditions in the LA-stroke group. The same results were found for the HA-stroke group (both paretic and non-paretic limbs) during standing on the rigid surface as well as in the control group during standing on the foam surface. However, during standing on the foam surface, a significant decrease of the CCI of both paretic and non-paretic limbs of the HA-stroke group was only observed in the HC condition. The greatest decrease of the CCI was observed in the HC condition (Fig 2A–2B).

**Table 5:** Summary of analysis of variance for co-contraction index (CCI) of the tibialis anterior and medial gastrocnemius muscles: F ratios, P values, and effect sizes by variable.

	CCI of the paretic limb (%)			CCI of the non-paretic limb (%)		
	F	P	$\eta_{p}^{2}$	F	P	$\eta_{p}^{2}$
**Main effect**						
Group	174.14	***<0*.*001***	0.88	268.83	***<0*.*001***	0.92
Condition	1500.63	***<0*.*001***	0.97	1655.78	***<0*.*001***	0.97
Standing surface	1860.36	***<0*.*001***	0.97	1268.28	***<0*.*001***	0.96
**Interaction effect**						
Group × Condition	63.45	***<0*.*001***	0.73	33.20	***<0*.*001***	0.58
Group × Standing surface	83.97	***<0*.*001***	0.78	40.67	***<0*.*001***	0.63
Condition × Standing surface	107.90	***<0*.*001***	0.69	463.52	***<0*.*001***	0.91
Group × Condition × Standing surface	35.87	***<0*.*001***	0.60	41.75	***<0*.*001***	0.63

**Table 6:** Simple main effects for analyzing inter-groups and inter-conditions differences of the co-contraction index (CCI) of the tibialis anterior and medial gastrocnemius muscles for all combinations.

				CCI of the paretic limb (%)		CCI of the non-paretic limb (%)	
				F	P	F	P
**Simple effect for analyzing inter-groups differences**							
**Standing surface**		**Rigid**	Baseline	136.06	***<0*.*001***	205.35	***<0*.*001***
			IF	129.66	***<0*.*001***	197.20	***<0*.*001***
			EF	85.64	***<0*.*001***	155.10	***<0*.*001***
			SC	73.45	***<0*.*001***	135.16	***<0*.*001***
			HC	43.48	***<0*.*001***	117.77	***<0*.*001***
		**Foam**	Baseline	242.60	***<0*.*001***	307.80	***<0*.*001***
			IF	228.14	***<0*.*001***	313.65	***<0*.*001***
			EF	220.41	***<0*.*001***	302.07	***<0*.*001***
			SC	234.88	***<0*.*001***	300.03	***<0*.*001***
			HC	200.44	***<0*.*001***	266.01	***<0*.*001***
**Simple effect for analyzing inter-conditions differences**							
**Group**	**Control**		Rigid	1.41	0.23	1.99	0.10
			Foam	20.82	***<0*.*001***	23.62	***<0*.*001***
	**LA-stroke**		Rigid	24.16	***<0*.*001***	14.43	***<0*.*001***
			Foam	46.32	***<0*.*001***	38.03	***<0*.*001***
	**HA-stroke**		Rigid	20.85	***<0*.*001***	17.10	***<0*.*001***
			Foam	20.94	***<0*.*001***	30.08	***<0*.*001***

IF, Internal focus; EF, External focus; SC, Simple cognitive task; HC, Hard cognitive task

## Discussion

This study was the first, to the best of the authors’ knowledge, to investigate the effects of anxiety and different strategies of directing attention (i.e. EF and cognitive task) on postural control of chronic stroke survivors compared with age-, sex-, height-, and weight-matched healthy subjects in terms of both postural sway measures and neuromuscular regulation. The results indicated that, during quiet standing on both rigid and foam surfaces, stroke survivors particularly those with a high level of anxiety, exhibited increased postural sway measures (i.e. decreased postural stability) compared with healthy subjects, which was accompanied by enhanced CCI at the ankle, leading to stiffening of the neuromuscular system. This reflects the cautious mode of postural control in stroke survivors especially in the HA-stroke survivors, which has a greater energetic cost and lower efficiency compared with the automatic mode of postural control observed in healthy subjects [53, 54]. Similar to healthy controls and LA-stroke group, distracting the attention away from postural control led to improved postural stability and decreased stiffening of the neuromuscular system in the HA-stroke group, but only under HC condition while standing on the foam surface and under EF, SC, and HC conditions while standing on the rigid surface.

The results showed greater postural sway measures (path length, SD of velocity along both ML and AP directions and mean velocity) of both LA- and HA-stroke groups (i.e. postural instability) in comparison with the healthy controls while standing on both rigid and foam surfaces, which was more evident in the HA-stroke group. Due to various stroke-induced sensory and motor impairments [2], the automaticity of postural control is reduced in stroke survivors, which increases postural instability [8]. Based on the attentional control theory, subjects with a high level of anxiety tend to direct their attention to movement control for compensating their attentional bias to task-unrelated stimuli [17]. Thus, anxiety may increase stroke-induced postural instability by interfering with automatic postural control processes. However, only during standing on the foam surface, the postural instability of the HA-stroke group was significantly greater than the LA-stroke group, implying that HA-stroke survivors are more prone to fall and its deleterious consequences than the LA-stroke survivors especially in the condition of the unstable standing surface.

Another important finding was that enhanced postural sway measures were accompanied by greater co-contraction of the ankle muscles of the paretic and non-paretic sides (i.e. TA and MGA) in both LA- and HA-stroke groups compared with the healthy group while standing on both rigid and foam surfaces, indicating regulation of postural control using stiffening strategy, which was more obvious in the HA-stroke group. Because of being energetically inefficient, this strategy is not an optimal strategy for quiet standing [55]. Using the stiffening strategy for postural control limits the flexibility of postural adaptations and hampers timely and proper responses to unexpected perturbations [56]. Houdjik et al. (2010) also reported greater co-contraction of TA and GA muscles of the non-paretic side in chronic stroke survivors than that of the healthy controls while standing on rigid and foam surfaces, which was associated with greater energy expenditure [57].

Further, the results revealed significantly greater co-contraction of ankle muscles of both paretic and non-paretic sides in the HA-stroke group as opposed to the LA-stroke group during standing on the foam surface, suggesting that stroke survivors who had a high level of anxiety may use improper increased cautious/conscious postural control processes while standing on unstable support surfaces. The resultant musculoskeletal stiffening may constrain the flexibility of postural adjustments, disturb appropriate responses to unexpected perturbations, and cause higher electrophysiological costs for postural control [48], possibly resulting in faster muscle fatigue and enhanced risk of fall in such situations [58]. Therefore, further attention should be paid to balance rehabilitation in HA-stroke survivors, not only to prevent falls but also to decrease fatigue and physical strain.

The current study also found that, in comparison with the rigid surface, standing on the foam surface led to a significant increase of postural sway measures in the three groups (i.e. LA-stroke, HA-stroke, and control groups), which was associated with a significant increase of ankle muscles co-contraction in the three groups. By decreasing sensory information (e.g. decreased proprioceptive information while standing on the foam surface), postural control becomes more difficult and needs more attentional resources [7, 49], leading to the enhanced use of the cautious mode of postural control and stiffening strategy. Previous studies also showed greater co-contraction of the ankle muscles during standing on the unstable support surface in healthy adults [52]. It has been suggested that ankle stiffening strategy may occur in an attempt to closely and consciously regulate the postural control because of postural threat (e.g. standing at height or on an unstable support surface) [59].

It is interesting to note that, in line with previous studies [9, 23, 24, 34], distracting the attention from postural control while standing on the rigid surface using the EF or cognitive tasks (both simple and hard) improved postural instability as compared with the baseline and IF conditions in the three groups as revealed by reduced postural sway measures. These changes in postural sway measures were accompanied by a significant decrease in CCI in the LA-stroke and HA-stroke groups. These results indicated that both EF and cognitive tasks engaged participants’ attention and provided less opportunity to consciously direct their attention to the postural control that has been reported to disrupt automatic postural control processes [60]. Contrary to the results of the current study, Negahban et al. [31] and Bensoussan et al. [32] reported increased postural sway of stroke survivors while standing quietly on the rigid surface and concurrently performing a cognitive task. The cognitive tasks used in these studies (i.e. Stroop task and arithmetic task, respectively) required vocal articulation, which may increase postural sway due to its related changes in breathing pattern and facial movements [61, 62]. Furthermore, during standing on the foam surface, significant improvement of postural stability and decreased neuromuscular stiffening was observed in the conditions of EF, and both SC and HC in the control and LA-stroke groups. However, in the HA-stroke group, the significant improvement of postural stability and decreased neuromuscular stiffening while standing on a foam surface was only found in the HC condition. This result suggests that during standing on unstable surfaces, distracting the attention only using a high demanding cognitive task can reduce the preoccupation of HA-stroke survivors with inefficient and energy-consuming conscious postural control.

Besides, the results showed that the cognitive tasks, especially the HC, improved postural stability more than EF in the three groups. One possible explanation for this may be that cognitive tasks need more complicated mental processes such as working memory, mental tracking, and decision making [63]. Thus, the cognitive tasks are more demanding compared with EF and may result in more distraction of attention from the postural control and allow the more efficient and automatized postural control processes to function in an unrestricted manner [26]. Moreover, the cognitive task may keep attention longer than EF, causing greater improvement of postural stability [23]. Previous studies also found that performing cognitive tasks led to enhanced postural stability of healthy young and older adults compared to both IF and EF conditions [23, 25].

Finally, some limitations need to be considered. First, among the muscles involved in postural control, we only assessed the ankle muscle activity. Previous studies have suggested two discrete control strategies (i.e. ankle strategy and hip strategy) for postural control. During quiet standing and small perturbations, the main strategy used for postural control is the ankle strategy. By increasing the difficulty of the postural task and/or in more perturbed situations, the use of hip strategy is usually increased [64, 65]. Given that the assessment of postural control in this current study was performed in the quiet standing, we focused on evaluating ankle strategy by measuring the muscle activity of the ankle (i.e. TA and MGA) similar to the previous studies [25, 66]. It is recommended that the evaluation of other muscles such as hip and trunk muscles be considered in future studies to provide further information about the postural control of chronic stroke survivors in different conditions. Second, investigating the effects of anxiety on postural control and muscle activity in chronic stroke survivors were limited to quiet standing. Future studies should aim to determine these effects when doing more complex and dynamic postural tasks (e.g. postural reactions to the perturbations of standing surface, walking on different surfaces) combining the quantitative kinematic analysis to attain a more comprehensive understanding of anxiety effects on static and dynamic postural control of chronic stroke survivors.

## Conclusions

The results of this study revealed that anxiety exacerbates stroke-induced internal focus on postural control and postural instability, promoting improper neuromuscular control of posture, with increased co-contraction of ankle muscles (i.e. ankle stiffening strategy), especially while standing on an unstable support surface. However, distracting the attention from postural control using EF or cognitive tasks could improve postural stability and decrease the use of inefficient stiffening strategy in chronic stroke survivors, even in those who had a high level of anxiety. An important implication of this study is that the effects of anxiety should be considered when evaluating postural control and implementing different interventions for improving postural control in chronic stroke survivors
